# Preface to theme issue about multi-messenger gravitational lensing

**DOI:** 10.1098/rsta.2024.0133

**Published:** 2025-04-10

**Authors:** Graham P. Smith, Martin A. Hendry, Federica Bianco

**Affiliations:** ^1^ School of Physics and Astronomy, University of Birmingham, Birmingham B15 2TT, UK; ^2^ Department of Astrophysics, University of Vienna, Türkenschanzstrasse 17, Vienna 1180, Austria; ^3^ SUPA, School of Physics and Astronomy, University of Glasgow, Glasgow G12 8QQ, UK; ^4^ Department of Physics and Astronomy, University of Delaware, 217 Sharp Lab, Newark, DE 19716, USA; ^5^ University of Delaware, Joseph R. Biden Jr. School of Public Policy and Administration, 184 Academy Street, Newark, DE 19716, USA; ^6^ University of Delaware Data Science Institute, Newark, DE, USA; ^7^ Vera C. Rubin Observatory, Tucson, AZ 85719, USA

**Keywords:** gravitational lensing, gravitational waves, black holes, neutron stars, neutrinos, gamma-ray bursts, supernovae, kilonovae, galaxies, galaxy clusters

Multi-messenger gravitational lensing combines multi-messenger astronomy with gravitational lensing. The first gravitational lensing observations occurred during the 1919 total solar eclipse, and were published in the *Philosophical Transactions of the Royal Society A*, thus providing early support for Einstein’s theory of General Relativity [1]. Systematic observations of gravitational lenses—beyond the Solar System and the Local Group—began decades later in the late twentieth century [2–4]. The ground-breaking detections of neutrinos from the Sun and SN1987A occurred on a similar timescale [5–9], marking the dawn of multi-messenger astronomy. This field experienced a spectacular renaissance three decades later when gravitational waves (GWs), and electromagnetic (EM) radiation from gamma rays to radio waves were detected from a binary neutron star (BNS) merger in 2017 [10,11]. The first confirmed discoveries of gravitationally lensed transient sources, and the first detection of neutrinos from an extragalactic source were both achieved in parallel with the early GW discoveries [12–14].

Multi-messenger gravitational lensing science spans a broad range of fundamental physics, cosmology and astrophysics that is central to many of the biggest open questions in physical science. In summary, this includes new experiments to explore the nature of gravity, our cosmological model including the expansion of the Universe, the nature of dark matter, when and how compact objects form, the physics of dense nuclear matter, the chemical enrichment of the Universe and the relationship between explosive transients and their host galaxies across cosmic time. In general, the complementary information that is available from a multi-messenger approach is central to enabling the science. Multi-messenger *gravitational lensing* adds an exciting new dimension, thanks to the sub-second timing accuracy of GWs, gamma rays and radio instruments. This points to a new class of gravitational lensing experiments that benefit from *both* sub-arcsecond sky positions *and* sub-second uncertainties on arrival time difference measurements of gravitationally lensed signals.

The breakthrough discoveries of 2015−2017 stimulated significant and rapidly growing interest in gravitational lensing of GWs and the role of multi-messenger techniques in their discovery and scientific applications [15–24]. This growing interest across diverse communities motivated us to propose a Royal Society Theo Murphy Discussion Meeting on the subject of Multi-messenger Gravitational Lensing. Our aim was to bring people together to review progress and to identify challenges and opportunities along the way to the first multi-messenger gravitational lensing discoveries. The meeting proposal was underscored by the broad consensus that current/upcoming instrument sensitivities are consistent with the first discovery happening within the next 5−10 years. It was therefore essential, and very well-timed to discuss questions such as ‘what do we as a diverse community need to do to recognise the lensed signals in the data streams from the respective instruments?’, ‘what can we learn from colleagues in closely related fields?’ and ‘what are the key scientific opportunities, and how might they shape the discovery effort?’*.*

The Theo Murphy meeting took place on 11−12 March 2024, at The Edwardian Manchester. As far as we are aware, the only previous meeting on this topic was ‘Einstein in Focus’, hosted by the Royal Astronomical Society in 2008, thus pre-dating the first direct detection of GWs by 7 years. This underlines the extraordinary progress achieved by many talented colleagues in the intervening years, including through the early direct detections of GWs and the imminent Legacy Survey of Space and Time (LSST) that will be conducted by the Vera C. Rubin Observatory (Rubin). The timing of the Theo Murphy meeting was especially prescient in the context of Rubin/LSST, as one week later the ‘Rubin ToO 2024’ workshop took place in Berkeley, including discussion of Rubin target of opportunity observations to follow-up sources including GWs (both lensed and not lensed), neutrinos and gamma-ray bursts (GRBs) [25].

All of the articles in this two-part Theme Issue are led by colleagues who gave presentations at the meeting. The articles describe multi-messenger gravitational lensing science, the state-of-the-art in searching for gravitationally lensed GWs and their lensed EM counterparts, lessons learned from related fields and tools and methods for discovery. The introduction to the whole Theme Issue was authored by a significant fraction of the meeting participants, and appears at the beginning of Part 2 [26]. In that introduction, we aim to provide an accessible introduction to the field for graduate students and more experienced researchers who are non-experts, including summaries of both the science cases for multi-messenger gravitational lensing and the challenges that the community aims to overcome in the next 3−5 years.

In Part 1, Matt Nicholl & Igor Andreoni [27] describe lessons learned from EM follow-up of GWs, Samantha Oates [28] gives the EM perspective on false positives when searching for counterparts to GW sources, Andrew Levan *et al*. [29] review the state-of-the-art in searching for gravitationally lensed GRBs, Ariel Goobar *et al*. [30] describe lessons learned from strongly lensed supernovae, Inés Pastor-Marazuela [31] provides the radio and fast radio burst (FRB) perspective on multi-messenger gravitational lensing, Arthur Offermans & Tjonnie Li [32] discuss applications of neural networks to identifying lensed GWs, Laura Uronen *et al*. [33] describes a multi-messenger approach to discovering gravitationally lensed binary black hole mergers and Luka Vujeva *et al*. [34] present the lenscat catalogue of known gravitational lenses.

Following the introduction, Part 2 continues with Otto Hannuksela’s [35] review of detection strategies for gravitationally lensed GW signals, then Dan Ryczanowski *et al*. [36] describes a strategy for discovering lensed EM counterparts to lensed GW sources that is running live in the fourth GW run, David Keitel [37] gives the GW perspective on false positives when searching for gravitationally lensed GWs, Christine Collins *et al*. [38] review how kilonova simulations connect observations with the underlying physics in the context of gravitational lensing, Simon Birrer *et al*. [39] review the challenges and opportunities for time-delay cosmography with multi-messenger gravitational lensing, Anupreeta More & Hemanta Phurailatpam [40] discuss progress towards combining the gravitationally lensed messengers and Anowar Shajib *et al*. [41] summarize the strong gravitational lenses that will emerge from Rubin/LSST in the next few years.

While these speakers and their articles cover a broad cross-section of topics relevant to multi-messenger gravitational lensing, we acknowledge that they are not complete. For example, despite best intentions, the constraints of a 2-day meeting led to some tough choices that resulted in relatively little emphasis at the meeting on neutrinos and variable sources. Also, scheduling constraints unfortunately prevented a few speakers from submitting an article.

Many important themes emerged from the Theo Murphy meeting. Some that caught our attention include the fact that the ultra-precise arrival time difference regime outlined above does not rely on the detection of gravitationally lensed GW signals. This helps to emphasize that significant observational progress can be achieved during the intervals between GW runs, for example, via studies that focus on discovering gravitationally lensed GRBs and FRBs. A further theme of broad relevance is the central role of gravitational lens catalogues for multi-messenger gravitational lensing discoveries—i.e. the synergy between the imminent several-order-of-magnitude expansion of strong lenses from Rubin and *Euclid* and the search for lensed binary compact object mergers via diverse messengers.

It was also very striking that different people define multi-messenger gravitational lensing in different ways. For example, some people prefer to concentrate on multiple messengers that come from the source that is lensed, while some prefer to include messengers that come from the host galaxy and/or lens itself in their definition. As organizers of the Theo Murphy meeting and Guest Editors of this Theme Issue, we choose not to prescribe one definition, and to value the different perspectives that everyone brings to the challenges and opportunities that we share.

To sum up, the overarching theme of the Theo Murphy meeting was the exciting scientific potential of multi-messenger gravitational lensing, the broad consensus that the first discovery is inevitable and could well occur within the next 5−10 years, and the need to get ready for that in the next 3−5 years. The future of multi-messenger gravitational lensing is bright!

## Data Availability

This article has no additional data.
